# Management of acute appendicitis during the COVID-19 pandemic: Views of two Brazilian surgical societies

**DOI:** 10.1590/0100-6991e-20202717

**Published:** 2021-09-28

**Authors:** JOSÉ GUSTAVO PARREIRA, LOUISIE GALANTINI LANA DE-GODOY, TERCIO DE-CAMPOS, PEDRO DE SOUZA LUCARELLI-ANTUNES, LUIZ GUSTAVO DE-OLIVEIRA-E-SILVA, HEITOR GAVIÃO SANTOS, RENATO ABRANTES LUNA, PEDRO EDER PORTARI, JOSE CESAR ASSEF

**Affiliations:** 1 - Faculdade de Ciências Médicas da Santa Casa de São Paulo, Departamento de Cirurgia - São Paulo - SP - Brasil; 2 - Irmandade da Santa Casa de Misericórdia de São Paulo, Serviço de Emergência - São Paulo - SP - Brasil; 3 - Irmandade da Santa Casa de Misericórdia de São Paulo, Departamento de Cirurgia - São Paulo - SP - Brasil; 4 - Hospital Federal de Ipanema, Serviço de Cirurgia Geral - Rio de Janeiro - RJ - Brasil; 5 - UniCETREx, Curso de Videocirurgia - Brasília - DF - Brasil; 6 - Hospital São Lucas, Departamento de Cirurgia - Brasília - DF - Brasil; 7 - Escola de Medicina e Cirurgia da UNIRIO, Disciplina de Cirurgia - Rio de Janeiro - RJ - Brasil; 8 - Hospital Federal do Estado do Rio de Janeiro, Serviço de Cirurgia Geral - Rio de Janeiro - RJ - Brasil; 9 - Hospital dos Servidores do Estado do Rio de Janeiro, Departamento de Cirurgia Geral - Rio de Janeiro - RJ - Brasil

**Keywords:** Emergencies, Pandemics, Coronaviridae Infections, Appendicitis, General Surgery, Emergências, Pandemias, Infecções por Coronaviridae, Apendicite, Cirurgia Geral

## Abstract

Acute appendicitis (AA) is a frequent cause of abdominal pain requiring surgical treatment. During the COVID-19 pandemic, surgical societies considered other therapeutic options due to uncertainties in the evolution of the disease. The purpose of this study is to assess the treatment of AA by members of two Brazilian surgical societies in this period. A common questionnaire was sent in 2020. There were 382 responses. Most surgeons had more than 15 years of profession (68.3%) and treated more than five cases per month (44.8%). About 72.5% would indicate chest CT to investigate COVID-19 in patients with AA. For those patients sustaining uncomplicated AA, without COVID-19, 60.2% would indicate laparoscopic appendectomy (VLA), followed by open appendectomy (OA) (31.7%) and non-operative management (NOM) (1.3%). For those with mild COVID-19, OA was suggested by 51.0%, followed by VLA (29.6%) and NOM (6.0%). For those with severe COVID-19, OA was proposed by 35.3%, followed by NOM (19.9%) and VLA (18.6%). For patients with periappendiceal abscesses, without COVID-19, VLA was suggested by 54.2%, followed by OA (33.2%) and NOM (4.4%). For those with mild COVID-19, OA was proposed in 49.5%, followed by VLA (29.3%) and NOM (8.9%). In those with severe COVID-19, OA was proposed in 36.6%, followed by NOM (25.1%) and VLA (17.3%). This information, based on two recognized Brazilian surgical societies, can help the surgeon to select the best approach individually.

## RESUMO

A apendicite aguda (AA) é causa frequente de abdome agudo cirúrgico. Durante a pandemia de COVID-19, devido às incertezas na evolução da doença, sociedades consideraram outras opções terapêuticas. Nosso objetivo é descrever o tratamento da AA por membros do CBC e SBAIT neste período. O questionário foi enviado em 2020. Houve 382 respostas. A maioria dos profissionais tinha mais de 15 anos de profissão (68,3%) e atendia mais de cinco casos por mês (44,8%). Cerca de 72,5% realizariam TC de tórax para investigação de COVID-19 em pacientes com AA. Nos com AA não complicada, sem COVID-19, 60,2% optariam pela apendicectomia videolaparoscópica (AVL), seguido de apendicectomia aberta (AAB) (31,7%) e tratamento não operatório (TNO) (1,3%). Nos com COVID-19 leve, AAB foi proposta por 51,0%, seguido da AVL (29,6%) e TNO (6,0%). Nos com COVID-19 grave, a AAB foi proposta por 35,3%, seguido de TNO (19,9%) e AVL (18,6%). Nos com AA complicadas com abscesso, sem COVID-19, AVL foi sugerida por 54,2%, seguida da AAB (33,2%) e TNO (4,4%). Nos com COVID-19 leve, a AAB foi proposta em 49,5%, seguidos da AVL (29,3%) e TNO (8,9%). Nos com COVID-19 grave, a AAB foi proposta em 36,6%, seguido de TNO (25,1%) e AVL (17,3%). Estas são opções de cirurgiões de duas sociedades cirúrgicas reconhecidas e podem auxiliar o colega que está na linha de frente a definir a melhor conduta individualmente.

## INTRODUCTION

The World Health Organization (WHO) considered the evolution of COVID-19 as a pandemic on March 11, 2020^1^. Since then, the spread has persisted, with profoundly serious consequences in several sectors. Parallel to this, non-traumatic surgical emergencies continue to be a frequent cause of acute abdominal pain in emergency services. Among these, acute appendicitis is one of the most frequent causes of surgically treated abdominal pain^2^ ^-^ ^4^. 

Operative treatment is considered the standard for most patients with acute appendicitis^4^. However, during the pandemic, non-operative treatment has been considered by several societies, based on arguments such as the worst evolution in patients who developed COVID-19 in the postoperative course and the resource allocation priority for the treatment of patients with COVID-19^5^ ^,^ ^6^. Studies demonstrated a higher incidence of respiratory complications, admission to intensive care, and deaths in patients undergoing elective surgeries who developed COVID-19 in the postoperative period^7^ ^,^ ^8^. Another factor is the possibility of contamination of the surgical team^9^.

Several publications have analyzed the non-operative treatment, with antibiotics and careful clinical observation, for patients with acute appendicitis prior to the onset of COVID-19^10^ ^-^ ^12^. There are authors who support the indication in uncomplicated appendicitis and even in cases of periappendicular abscesses. Current consensus places this form of treatment as optional in some cases^4^. Despite this support in the literature, it cannot be said that the use of NOM in patients with acute appendicitis and COVID-19 is equally safe. NOM protocols were developed in different contexts, in which there is no infection concomitant with the abdominal condition.

We do not know exactly how SARS-COV-2 interferes with the evolution of patients with intra-abdominal infections. There are no studies with appropriate methodology that allow the comparison of the evolutions of patients treated in different ways. Our hypothesis is that the evaluation of the options used among Brazilian surgeons for the treatment of patients with acute appendicitis during the pandemic period can help us make decisions in critical cases. The aim of this study is to perform a descriptive analysis of the options most used by Brazilian surgeons in the treatment of patients with acute appendicitis, as well as to understand the impact of the pandemic on this approach.

## METHODS

 After a literature review on the subject, the authors discussed the most important topics. The open doubts were put in the form of questions, selected to remain faithful to the proposed theme. At the end of the process, we chose eleven questions, whose content included practice time as a surgeon, the number of patients with acute appendicitis treated monthly, and therapeutic aspects.

We stratified the severity of acute appendicitis into two groups:

- Uncomplicated acute appendicitis;
- Acute appendicitis complicated with abscess.

We addressed the treatment of these conditions in four clinical situations:

- Before the COVID-19 pandemic;
- During the COVID-19 pandemic, in patients with no suspected disease (COVID-19 negative clinical picture and chest tomography);
- COVID-19 positive or suspected patients with mild forms of the disease;
- COVID-19 positive or suspected patients with severe forms of the disease (requiring mechanical ventilation).

We also added one question related to performing chest tomography as a method for identifying COVID-19 pneumonitis in patients with acute appendicitis.

This questionnaire was structured with the aid of the tool Survey Monkey® (www.surveymonkey.com) and Google Docs®. We chose two Brazilian medical societies (Brazilian College of Surgeons - CBC, and Brazilian Trauma Society - SBAIT) as the basis of this research. The questionnaire was independently sent by email to CBC members and via Whatsapp app to SBAIT physicians in June 2020. Physicians were asked to respond only once. After seven days, we closed the acquisition of data, which were descriptively evaluated. We compared responses at times before and during the pandemic, as well as between patients’ clinical conditions.

## RESULTS

**Figure 1:**
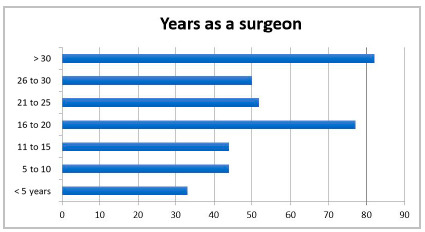
Time of professional experience as a surgeon (in years).

**Figure 2:**
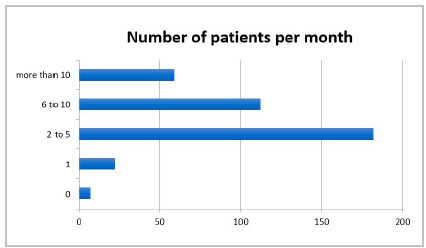
Surgeon experience: Number of patients sustaining appendicitis treated per month.

We received 382 responses, 350 from CBC members and 32 from SBAIT ones. We observed that most surgeons who answered the questionnaire had been in practice for more than 15 years (68.3%). The length of surgical experience was balanced in the analyzed intervals (every 5 years), with a predominance of the ranges from 16 to 20 years (20.1%) and more than 30 years (21.5%) (Figure 1). Approximately 44.8% of the respondents reported treating more than 5 cases of acute appendicitis per month (Figure 2).

**Figure 3:**
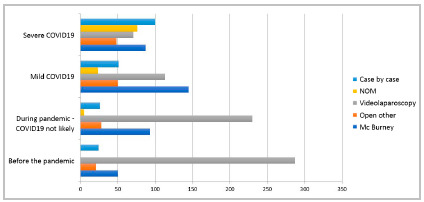
Management of patients with uncomplicated appendicitis in four situations: before the pandemic, during the pandemic (patient with no symptoms or signs of COVID19), patients with diagnosis of mild COVID19 and patients with severe COVID19. NOM= Nonoperative management, Open other = any open laparotomy other than Mc Burney, Case by case = decision was made case by case..

**Figure 4:**
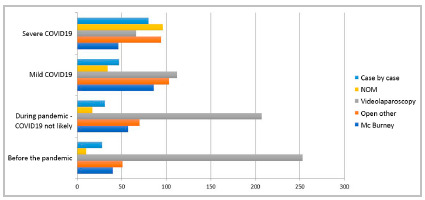
Management of patients with complicated appendicitis (abscess) in four situations: before the pandemic, during the pandemic (patient with no symptoms or signs of COVID-19), patients with diagnosis of mild COVID-19 and patients with severe COVID-19. NOM= Nonoperative management, Open other = any open laparotomy other than Mc Burney, Case by case = decision was made case by case.

Before the pandemic, the most frequently proposed treatment was laparoscopic appendectomy, both for uncomplicated appendicitis (75.1%) and for cases complicated with local abscess (66.2%). Together, the other therapeutic options did not exceed 35.0% of the total. The open route was proposed for 18.6% of patients with uncomplicated appendicitis and for 23.8% with complicated forms. No surgeon opted for non-operative treatment for patients with uncomplicated acute appendicitis and in those with appendicitis complicated with abscess, NOM was suggested by 2.6% (Figures 3 and 4).

Approximately 72.5% of surgeons would indicate chest tomography for the investigation of COVID-19 in patients with acute appendicitis, with 39.8% indicating it for all patients and 32.7%, selectively.

For the treatment of patients with uncomplicated acute appendicitis during the pandemic but without suspected COVID-19, laparoscopic appendectomy was suggested by 60.2%, followed by open approach (31.7%) and NOM (1.3%) (Figures 3 and 4). In COVID-19 positive or suspected patients with mild disease, the open route was proposed in 51.0%, followed by laparoscopy (29.6%) and NOM (6.0%). In cases of positive or suspected COVID-19 in the severe form, the open route was proposed in 35.3%, followed by NOM (19.9%) and laparoscopy (18.6%). Individualized treatment (case-by-case definition) was suggested for patients without suspected COVID-19 in 6.8%, in those with mild COVID-19 in 13.3%, and in those with severe form in 26.2%.

For the treatment of patients with acute appendicitis complicated with abscess during the pandemic, in cases without suspicion of COVID-19, the laparoscopic route was suggested by 54.2%, followed by the open route (33.2%) and NOM (4.4%) (Figures 3 and 4). In COVID-19 positive or suspected patients with the mild form, the open route was proposed in 49.5%, followed by laparoscopy (29.3%) and NOM (8.9%). In cases of suspected or confirmed COVID-19 in the severe form, the open route was proposed in 36.6%, followed by NOM (25.1%), and the laparoscopic route (17.3%). Individualized treatment (case-by-case definition) was suggested for 8.1% of patients with no suspicion of COVID-19, 12.3% for those with COVID-19 in the mild form, and for 20.9% in the severe form.

## DISCUSSION

In 2018, Sartelli et al. evaluated the treatment of acute appendicitis in a prospective observational study with 4,282 patients^13^. Of these, approximately 95.7% were surgically treated, 51.7% laparoscopically and 42.2% with open surgery. In that study, 4.3% of cases were treated non-operatively.

In our research, we observed that, in the pre-pandemic period, laparoscopic appendectomy was the most frequently suggested option for the treatment of patients with acute appendicitis, regardless of the presentation (75.1% in uncomplicated forms and 66.2% in complicated ones). It is important to note that NOM was not an option in the uncomplicated forms. Even in cases complicated with abscesses, there was no prominent place for NOM (2.6%) in our country. Compared with international data, we observed a higher frequency of the laparoscopic approach, with a lower indication for NOM.

Ielpo et al. published a study in 2020 assessing the conduct in acute appendicitis during the pandemic through questionnaires sent to surgeons in 66 countries^14^. There were 709 responses. They observed that non-operative treatment increased during the pandemic period, both in cases of uncomplicated (2.4% to 5.3%) and in complicated (6.6% to 23.7%) acute appendicitis; approximately a third of surgeons opted for open surgery over laparoscopic. We also noticed this trend in our data, the open route being indicated more frequently in COVID-19 positive cases. This finding agrees with the current literature, since the open approach was suggested by other authors in an attempt to reduce the risk of viral transmission to the team^5^ ^,^ ^9^.

During the COVID-19 pandemic, several surgical societies proposed NOM as one of the forms of treatment for patients with acute appendicitis, based on factors such as the risk of contamination of the surgical team by SARS-COV-2, the unpredictable evolution from the point of view of the respiratory condition in patients undergoing surgical treatment, and the allocation of resources to the treatment of patients with COVID-19. Thus, NOM emerged as an interesting option, being included in the treatment protocols of some centers^15^.

Podda et al. (2021) identified nine studies that reported non-operative treatment in patients with acute appendicitis during the pandemic^15^. The frequency of NOM ranged from 7.8% to 91.0% of cases. The largest of these series was published by Bassamh et al., in 2020, analyzing 42 patients treated non-operatively during the pandemic period^6^. Of these, six (14.3%) required laparoscopy and/or laparotomy further on, one required percutaneous drainage, and two, change in the antibiotics’ regime. This failure frequency is comparable to that observed in pre-pandemic studies^10^ ^,^ ^11^. In our study, even for patients with confirmed or suspected COVID-19, NOM was not a frequent option, except for cases of complicated appendicitis and severe COVID-19, reaching approximately 25% of responses. This may be related to the fact that the surgeons did not routinely apply NOM outside the pandemic period, as also observed in our study. However, in the complicated forms of COVID-19, the option of “case-by-case assessment” has grown significantly, opening the possibility of applying NOM in selected cases.

Concern about the identification of patients with COVID-19 was clear, since over 70% of surgeons would indicate chest tomography in patients with acute appendicitis. This shows the importance that the surgeon gives to this clinical condition, both due to the risk of transmission to the team and to the possibility of worsening the clinical picture. 

We observed that the open route was progressively used in cases of patients with suspected or positive COVID-19. This can be explained by the surgeon’s concern with the worsening of the respiratory condition, as well as the possibility of transmission to the surgical team. Open McBurney incision appendectomy can be performed with spinal/epidural anesthesia, avoiding the risks related to tracheal intubation, as well as pressure ventilation in a patient with pneumonitis. However, the laparoscopic approach is not contraindicated in patients with COVID-19 if the patient supports the pneumoperitoneum and specific care is taken. Furthermore, the risk of contamination must also be considered in the open route, as it is believed that the virus can remain viable in the smoke from the electric scalpel^5^.

For both patients with uncomplicated acute appendicitis and those with acute appendicitis with periappendicular abscesses, we proposed three clinical situations to be analyzed: patients without suspected COVID-19, mild suspected/positive COVID-19, and severe suspected/positive COVID-19. In the comparison of these six scenarios, the decrease in use of the laparoscopic route is remarkable, as well as the increase in the open route, NOM, and case-by-case definition as the severity of appendicitis and COVID-19 increases. We believe that this reflects the growing complexity of the cases, in association with the real possibility of contamination of the team. It is important to emphasize that, when excluding the “case-by-case” decision, we noticed that, in all clinical situations proposed, surgical options were the most frequent, regardless of the access route.

We did not include the clinical situation of “diffuse peritonitis” in the questionnaires, as we believe that in this circumstance, the surgical indication is necessary. However, our study demonstrates the variability of therapeutic options for the most common cause of acute abdomen during the pandemic period. Importantly, this may be related to many factors, including the availability of supplies and surgical equipment, surgeon training, and local conditions. Regardless, the pandemic has clearly changed the treatment of patients with acute appendicitis as most surgeons understand it. This should be a wake-up call to everyone, as we are at a time when different parts of our country are experiencing their own realities regarding the pandemic.

It is worth noting the limitations of this analysis. The sample of 382 surgeons may not be adequate, as there was no sample calculation. Due to problems related to databases, we were unable to cross-reference more data for statistical analysis. Perhaps the approaches would be different had we compared more seasoned surgeons with less experienced ones, for example. There is a lot of variability in the availability of materials for laparoscopy in a continental country like Brazil. Therefore, the option for the open route, more than a technical option, may reflect the lack of specific material in public services.

Despite the limitations, this study emerges as a support tool for the surgeon who is on the front line. We chose this publication, even with limitations, as the data collected here are informative and descriptive. Time is pressing. We need to make tools available to assist decision making in critical moments such as the one we are living. While we do not have prospective studies on the subject, the information from this research can serve as a basis for other Brazilian surgeons, exposing the thinking of an experienced group from two recognized national societies.

In conclusion, the final analysis of the data indicates that, during the COVID-19 pandemic, preoperative chest tomography assessment is performed by most surgeons, even in asymptomatic patients. For patients without suspected COVID-19, laparoscopic appendectomy remained the first option. In those with suspected or confirmed COVID-19, open appendectomy gains space, both in complicated and uncomplicated appendicitis. Non-operative treatment was proposed only by a small portion of surgeons, being more frequent in cases of appendicitis complicated with abscess in patients with severe COVID-19 (close to 25% of cases).

## References

[1] WHO (2020). WHO announces COVID-19 outbreak a pandemic [Internet].

[2] Cervellin G, Mora R, Ticinesi A, Meschi T, Comelli I, Catena F (2016). Epidemiology and outcomes of acute abdominal pain in a large urban Emergency Department retrospective analysis of 5,340 cases. Ann Transl Med.

[3] Viniol A, Keunecke C, Biroga T, Stadje R, Dornieden K, Bösner S (2014). Studies of the symptom abdominal pain--a systematic review and meta-analysis. Fam Pract.

[4] Di Saverio S, Podda M, De Simone B, Ceresoli M, Augustin G, Gori A (2020). Diagnosis and treatment of acute appendicitis 2020 update of the WSES Jerusalem guidelines. World J Emerg Surg.

[5] COVIDSurg Collaborative (2020). Global guidance for surgical care during the COVID-19 pandemic. Br J Surg.

[6] Basamh M, Rajendiran A, Chung WY, Runau F, Sangal S (2020). Management of appendicitis during the COVID pandemic lessons from the first month of the outbreak. Br J Surg.

[7] Lei S, Jiang F, Su W, Chen C, Chen J, Mei W (2020). Clinical characteristics and outcomes of patients undergoing surgeries during the incubation period of. COVID-19 infection, EClinicalMedicine.

[8] COVIDSurg Collaborative (2020). Mortality and pulmonary complications in patients undergoing surgery with perioperative SARS-CoV-2 infection an international cohort study. Lancet.

[9] Veziant J, Bourdel N, Slim K (2020). Risks of viral contamination in healthcare professionals during laparoscopy in the Covid-19 pandemic. J Visceral Surg.

[10] Podda M, Gerardi C, Cillara N, Fearnhead N, Gomes CA, Birindelli A (2019). Antibiotic treatment and appendectomy for uncomplicated acute appendicitis in adults and children a systematic review and meta-analysis. Ann Surg.

[11] Salminen P, Paajanen H, Rautio T, Nordström P, Aarnio M, Rantanen T (2015). Antibiotic therapy vs appendectomy for treatment of uncomplicated acute appendicitis the APPAC randomized clinical trial. JAMA.

[12] Simillis C, Symeonides P, Shorthouse AJ, Tekkis PP (2010). A meta-analysis comparing conservative treatment versus acute appendectomy for complicated appendicitis (abscess or phlegmon). Surgery.

[13] Sartelli M, Baiocchi GL, Di Saverio S, Ferrara F, Labricciosa FM, Ansaloni L (2018). Prospective Observational Study on acute Appendicitis Worldwide (POSAW). World J Emerg Surg.

[14] Ielpo B, Podda M, Pellino G, Pata F, Caruso R, Gravante G (2020). Global attitudes in the management of acute appendicitis during COVID-19 pandemic: ACIE Appy Study. Br J Surg.

[15] Podda M, Pata F, Pellino G, Ielpo B, Di Saverio S (2021). Acute appendicitis during the COVID-19 lockdown never waste a crisis!. Br J Surg.

